# Social support and life-threatening behavior in online psychotherapy patients: a possible “pulling-together effect” during COVID-19

**DOI:** 10.1186/s41155-026-00384-3

**Published:** 2026-03-20

**Authors:** Cleonice Zatti, Neusa Sica da Rocha

**Affiliations:** 1 Graduate Program in Psychiatry and Behavioral Sciences, Universidade Federal do Rio Grande do Sul, Ramiro Barcelos, 2400, 2nd floor, Porto Alegre, RS 90035-003 Brazil; 2 Center of Clinical Research, Hospital de Clínicas de Porto Alegre, Porto Alegre, RS Brazil; 3 Innovations and Interventions for Quality of Life Research (I-QoL), Porto Alegre, RS Brazil

**Keywords:** Pulling-together effect, Life-threatening behavior, Social relationships, Psychotherapy

## Abstract

**Background:**

The “pulling-together effect” has been widely studied in the context of large-scale crises, such as wars, natural disasters, epidemics, and pandemics. This study was conducted from November 2020 to June 2021 and suggests a possible association with this effect, as this period may represent one of the most intense phases of the pulling-together effect ever studied in the context of global catastrophes.

**Objective:**

This study aimed to evaluate the association between life-threatening behavior and satisfaction with social relationships in online psychotherapy patients, which may be related to the pulling-together effect and the COVID-19 pandemic.

**Method:**

The clinical sample was composed mostly of females (80%), aged from 21 to 30 years. Patients were selected from the psychotherapy services of five major hospitals in Porto Alegre, a state capital in southern Brazil.

**Results:**

Preliminary results indicated that 41 individuals (44%) had some form of life-threatening behavior, and 62 (66%) rated their satisfaction with personal relationships as satisfactory or indifferent. After adjustment for other variables—gender, skin-color, and marital status—satisfaction with social relationships showed a protective effect, reducing life-threatening behavior by 29% (95%CI: 0.59–0.86, *p* < 0.001).

**Conclusions:**

Our results suggest that satisfaction with social relationships was protective against suicidal behavior in the early stages of the pandemic. In this sample, we identified a possible association with the pulling-together effect.

**Supplementary Information:**

The online version contains supplementary material available at 10.1186/s41155-026-00384-3.

## Introduction

The “pulling-together effect” has been widely studied in the context of large-scale crises, such as wars, natural disasters, epidemics, and pandemics. The hypothesis is that when individuals from the same group share similar experiences, they tend to provide mutual support by their social relationships (MS., 2021). This theory has been discussed by authors for several decades; some authors argue that severe suicidal behavior is associated with an increased need for belonging (Joiner et al., 2006; Baumeister & Leary, 1995). Belonging to a group or community can be so powerful that this emotional experience, or the perception of it, may trigger protective processes against suicide, even when risk factors are involved (Aran et al., 2023; Gutierrez et al., 2022).

Periods of national tragedy are often accompanied by negative aspects and detrimental effects on mental health; however, individuals who manage to unite or experience a sense of belonging to a group may contribute to reducing suicide rates (Mournet et al., 2022; Joiner et al., 2006). The pulling-together effect has been associated with positive mental health outcomes, as it not only helps reduce suicide risk but also reduces depressive symptoms (Levi-Belz & Feigelman, 2022). Hence, human connections seem to function as a “safety net,” helping to protect individuals from suicidal risk.

At the beginning of 2020, the world’s population faced a pandemic caused by the COVID-19. The slogan “stay at home,” came to symbolize a pause in everyday life, creating an unprecedented scenario in Brazil and in the world (Bosi & Alves, 2023). During this period, concerns related to illness and death intensified, and suffering was experienced both within households and in psychotherapy settings. Several protective measures were adopted, including the transition from in-person psychotherapy to online modalities, also known as e-therapy. These selective and universal interventions contributed to suicide prevention by increasing access to existing psychotherapy treatments (Gunnell et al., 2020).

Suicidal behavior can take various forms, including: suicidal ideation (fantasizing about being dead or thinking about dying), planning (developing a suicide plan following ideation), suicide attempts (non-lethal self-injury with intent to die), and suicide (self-inflicted death) (Botega, 2017). This behavior is primarily defined as a preoccupation with, desire for, or act of causing self-harm. When present, it is considered a serious condition and requires trained professionals to develop strategies for support, early identification, treatment of vulnerable populations, and the implementation of measures to prevent suicidal behavior (Miranda et al., 2024). Particularly in the context of a pandemic, a key challenge for mental health professionals is to support individuals in constructing meaning in their lives—having a sense of purpose and direction—when facing adversity (Zatti et al., 2024).

Data on suicide rates in the United States showed a decrease during the first months of the COVID-19 pandemic, suggesting the presence of a pulling-together effect (MS., 2021). In this effect, individuals tend to perceive themselves as sharing the same crisis, which increases their sense of group belonging and may lead to a reduction in the risk of suicidal behavior. A similar pattern occurred in Japan at the beginning of the pandemic, in which suicide rates also declined, suggesting a temporary increase in social cohesion and further supporting the pulling-together effect (Nakanishi et al., 2021).

However, in wartime, this effect tends to dissipate once the conflict ends, leaving individuals feeling disappointed and deeply helpless in the face of devastated territories. Such experiences may lead to feelings of discouragement, abandonment, and defeat, depleting personal resources due to prolonged daily struggle and resulting in chronic stress associated with trauma that can persist for months or even years (Ferracioli et al., 2021). Similarly, after the second wave of the pandemic in Japan, the attenuation of the effect was followed by pandemic fatigue and suffering related to social deprivation, which coincided with an increase in suicide rates (37,180 suicides over 21 months of the pandemic) (Nakanishi et al., 2021).

A study conducted in southern Brazil found stability in the overall incidence of suicide deaths after the onset of the pandemic, compared with projected estimates. The stratified analysis revealed differences across the evaluated groups, including age, gender, educational level, and skin color). Despite the overall stability in suicide numbers, the number of people with a history of limited access to health services increased and may have been disproportionally affected by the pandemic (Ornell et al., 2022).

Individuals affected by social deprivation and confinement during a pandemic may develop psychological symptoms. A study involving 3,274 participants showed that most individuals who met the criteria for depressive episodes reported low levels of social support, spirituality, resilience, quality of life, physical exercise, and educational attainment—factors considered predictors of depressive symptoms during COVID-19 quarantine (Schmitt et al., 2021).

As the pandemic posed a potential risk to mental health (Schmitt et al., 2021), psychotherapists from several theoretical lines evaluated the pandemic context, and online or home-based remote services emerged as alternatives to minimize the pandemic harmful effects while maintaining psychological care. With the onset of the pandemic, the adoption of this modality accelerated significantly, driven by the need to sustain ongoing treatments and to respond to increased psychological distress (Londero et al., 2021). This shift marked the beginning of a new mode of clinical practice: via online appointments, many patients were able to address conflicts and anxiety, potentially preventing depression, stress, and post-traumatic stress disorder in the long term.

COVID-19 was not an experience confined to hospital settings but a life event that affected nearly everyone’s daily routines. This traumatic context escalated quickly in both work and home environments, and reports of fear and concern consequently became prominent in psychotherapy sessions. Such sessions shifted to a two-dimensional format via the internet, posing a new challenge for both therapists and patients, whose work had traditionally been grounded in in-person settings. The therapeutic alliance became a concern for many therapists, who questioned how it could be established in an online contexts and feared that expressions of empathy, sensitivity, and control over the therapeutic setting might be compromised (Schmidt et al., 2020). However, mental health professionals who used the internet as a clinical tool gradually observed that the therapist–patient dyad could still experience a “three-dimensional affective presence” through the screen—a depth of connection previously associated only with face-to-face encounters.

The implementation of more accessible therapeutic interventions during the pandemic, such as online care, reflects the ability of technology to overcome geographical barriers in mental health services, proving relevant for individuals with severe mental disorders living in both remote and urban areas. The pandemic has had numerous impacts on the population, including emotional difficulties and disruptions in social relationships. In light of these factors, it is important to discuss such issues to help prevent behaviors detrimental to health (Londero et al., 2021). Thus, this study aimed to evaluate the association between life-threatening behavior and satisfaction with social relationships among online psychotherapy patients, which may be related to the pulling-together effect and the pandemic.

Based on these data, this study used a sample from the project entitled “Online Psychotherapies.” This umbrella project evaluated patients from five psychiatric training and reference institutions in southern Brazil, constituting a naturalistic, longitudinal, and online design.

The study was conducted by the Hospital de Clínicas de Porto Alegre, Brazil, registered on Plataforma Brazil (CAAE: 31680320700005327). It was approved in 2020 by the Research Ethics Committee of Hospital de Clínicas de Porto Alegre (approval No. 2020 − 0242). All participants provided informed consent.

## Methods

### Participants

Patients from five psychotherapy services in the city of Porto Alegre were invited to participate in the data collection as follows: 32 from the Hospital de Clínicas de Porto Alegre (HCPA); 17 from the Fundação Mario Martins (FUMM); 12 from the Hospital Nossa Senhora Conceição (HNSC); 21 from the Hospital São Pedro (HSP); and 12 from the Centro de Estudos Luís Guedes (CELG). Participants were selected by consulting the psychotherapy services of five major hospitals in Rio Grande do Sul State, Brazil. All patients were receiving psychotherapy provided by second- and third-year psychiatry residents. Data were collected using a Survey Monkey link sent to eligible patients. In total, 148 individuals completed the survey. After applying the inclusion criteria (being 18 years old or over and current engagement in psychotherapy) and exclusion criteria (inability to understand or complete the questionnaire [*n* = 19] or incomplete responses on the analyzed instruments [*n* = 35]), a final sample of 94 patients were included.

### Instruments

This study is part of a broader multicenter research project whose protocol included several instruments. For the purposes of this analysis, we used: sociodemographic data, the Patient Health Questionnaire-9 (PHQ-9) (Santos et al., 2013), and the EUROHIS-QOL 8-item index (Pires et al., 2018). Data were collected from November, 2020 to June, 2021.

### Data analysis

Only one item of each instrument was selected, aiming to compose a cross-sectional analysis within a longitudinal study. The selected PHQ-9 item asks patients: “Over the last two weeks, many days did you had thoughts that you would be better off dead or of hurting yourself in some way?” This item was used as an indicator of life-threatening behavior. The selected item from the EUROHIS-QOL 8-item—“How satisfied are you with your personal relationships (friends, relatives, acquaintances, colleagues)?”—was used to assess satisfaction with personal relationships.

### Statistical analysis

Proportions were compared using the Chi-squared test. To estimate prevalence ratios (PRs), robust Poisson regression was applied. A multivariable analysis was conducted to verify the independent association between satisfaction with social relationships and life-threatening behavior. The statistical analyses are justified by the dichotomization of the outcome variable (suicidal behavior) as follows: *“phq9_positive 1 ‘someday during the last 15d’ 0 ‘none’*.*”* Data were analyzed using SPSS 18, and statistical significance was set at p = 0.05.

## Results

The clinical sample was composed mostly of females (80%), aged 21 to 40 years (Table 1). Analysis of the sample indicated that 41 individuals (44%) reported some form of life-threatening behavior, and 62 (66%) rated their satisfaction with personal relationships as satisfactory or indifferent. The prevalence ratio between satisfaction with social relationships and suicide risk was 0.74 (95%CI: 0.61–0.89, *p* = 0.002). This ratio indicates that satisfaction with social relationships was associated with a 26% reduction in life-threatening behavior.

**Table 1:** Sample characteristics of online psychotherapy patients from five hospital-based services in southern Brazil

Characteristic	Total			PR (95%CI)
	*N*	%		
Total	94	100%		
Male	19	20%	6 (31.6%)	0.68 (0.33;1.37)
Female	75	80%	35 (46.7%)	1.00
Up to 20 years old	2	2	1 (50.0%)	2.00 (0.22;17.9)
21–40 years old	54	57	27 (50.0%)	2.00 (0.36;11.1)
41–60 years old	34	36	12 (35.3%)	1.41 (0.24;8.18)
Over 61 years old	4	4	1 (25.0%)	1.00
Incomplete high school	13	14	6 (46.2%)	1.98 (0.83;4.75)
Complete higher education or high school degree	51	54	28 (54.9%)	2.35 (1.18;4.71)
Bachelor’s degree with or without graduate degree	30	32	7 (23.3%)	1.00
Skin-color				
White	74	79%	30 (40.5%)	0.74 (0.46;1.20)
Black	20	21%	11 (55.0%)	1.00
Marital status				
Unmarried	53	56%	26 (49.1%)	1.34 (0.82;2.18)
Married	41	44%	15 (36.6%)	1.00
Occupation				
Employed	38	40%	11 (28.9%)	1.00
Student	21	22%	10 (47.6%)	1.65 (0.84;3.22)
Retired/health insurance	18	19%	11 (61.1%)	2.11 (1.14;3.92)
Unemployed	13	14%	7 (53.8%)	1.86 (0.92;3.78)
Homemaker	4	4%	11 (28.9%)	1.73 (0.58;5.19)

Unmarried individuals are those who are single (never married), separated, divorced, widowed

*PR* Prevalence ratio, *CI* Confidence interval

Among the 94 patients included in the sample, 79% (*n* = 74) self-identified as white, and 57% (*n* = 53) did not have a permanent partner. Regarding educational attainment, 86% (*n* = 81) had completed high school or higher education. Among men (*n* = 19), 32% (*n* = 6) reported suicidal behavior, whereas among women (*n* = 75), 47% (*n* = 35) reported such behavior, representing a 15% difference between the groups. Although not statistically significant, this finding shows that women presented higher levels of life-threatening behavior (*p* = 0.355). Among non-white individuals (*n* = 11), 55% reported risk behavior or suicidal ideation, compared with 40.5% (*n* = 30) among self-identified white individuals; this difference was not statistically significant (*p* = 0.367). Regarding marital status, 49% (*n* = 26) of the participants without a permanent partner reported suicidal behavior, compared with 37% (*n* = 15) of those with a steady partner 37% (*n* = 15). Thus, unmarried individuals showed a higher risk of self-destructive behavior (*p* = 0.318).

After adjustment for other variables—gender, skin-color, and marital status—the association between satisfaction with social relationships and life-threatening behavior yielded a 0.71 prevalence ratio (95%CI: 0.59–0.86, *p* < 0.001). Therefore, satisfaction with social relationships was associated with a 29% reduction in the prevalence of life-threatening behavior.

## Discussion

This study aimed to analyze the association between life-threatening behavior and satisfaction with social relationships among individuals receiving online psychotherapeutic treatment during the COVID-19 pandemic. We confirmed the initial hypotheses, as satisfaction with social relationships was a protective factor against suicidal behavior in the early stages of the pandemic. Social relationships showed a protective effect, reducing the risk of developing life-threatening behavior by 29%.

Perceived social support is a well-established protective factor against suicidal ideation. It is believed that a lack of belonging and perceptions of being a “burden” within social contexts increases the risk of suicidal ideation (Mournet et al., 2022; Zatti et al., 2018). Although social support can be understood as a “free” and publicly available resource, it is not equally accessible to all individuals. In more severe cases—such as suicide attempts in the absence of family or social support for post-hospitalization follow-up—readmission rates tend to increase. Therefore, health systems must actively monitor high-risk patients after hospital discharge or following a suicide-related crisis. Psychotherapy treatment should be routinely implemented, as it has also been shown to significantly reduce suicide attempt rates (Mann et al., 2021).

Although we reviewed the scientific literature on the pandemic period, we are not aware of studies that have specifically addressed the interaction between these factors and the pulling-together effect. This effect has been discussed across different regions and historical contexts, and substantial evidence has documented its preventive influence on suicide risk following disasters. For example, after the terrorist attacks of 9/11 in the United States, in which sociological forces significantly reduced suicide rates, with a uniform and temporary decline in rates neighboring the World Trade Center (Claassen et al., 2010). Similarly, decreases in suicide rates during periods of war appear to follow this same pattern (Rojcewicz, 1971).

We highlight the importance of understanding this phenomenon, even though it is cross-sectional, as it suggests that the effect may emerge in contexts of catastrophes or pandemics. Social forces can lower suicide rates; nonetheless, social support functions as a prevention tool only when individuals actually receive the support they seek (Mournet et al., 2022).

Data collection from November 2020 to June 2021 suggests a possible association with the pulling-together effect, as this period may be one of the most intense phases of this effect ever studied in the context of global catastrophes.

Could these findings represent preliminary evidence of the pulling-together effect during the initial stages of the COVID-19 pandemic? To address this question more conclusively, longitudinal studies that repeatedly evaluate changes in suicide mortality rates before, during, and after the pandemic are much needed. Such designs could provide information with important implications for the development of preventive interventions in future large-scale crises.

As a hypothesis, if the pulling-together effect was already experienced during the initial months of the pandemic, concerns emerge regarding the period following its dissipation. Although this effect may exert a temporary protective role, it is not permanent. Evidence from wartime contexts suggests that the pulling-together effect often diminishes after the conflict ends, followed by increased vulnerability associated with prolonged stress and collective disappointment (Ferracioli et al., 2021).

Brazil registered 10,409 suicides from March to December 2020, representing an overall reduction of 13% compared to the estimated rates for the same period (Orellana et al., 2022). However, from 2021 onwards, suicides rates increased disproportionately in specific populations: a 26% excess among men in the North and a 40% excess among women in the Northeast—regions historically marked by greater socioeconomic inequality (Orellana et al., 2022). In these regions, the difference in suicide prevalence between men and women was approximately 14%. In our sample, suicidal behavior was more prevalent among women, with a comparable gender difference of 15% between the two groups.

After analyzing the data and developing this study, we acknowledged the importance of discussing these findings and their implications for Brazilian public health. Certain population groups were disproportionately affected by the social isolation during the pandemic. Women, in particular, experienced increased emotional overload due to the accumulation of daily roles, which intensified during this period. Among other vulnerable groups—although not directly examined in this study but relevant to acknowledge—are the LGBTQIA+ population and older adults. The former is routinely exposed to discrimination, social stigma, lack of belonging, and violence, factors intensified during the pandemic (Ethier et al., 2024). The latter, especially those with limited social support or facing physical and emotional barriers to community life, experienced heightened loneliness and weakened feelings of belonging (Kim et al., 2024). Taken together, these factors constitute a major public health challenge in Brazil, as they contribute to high suicide rates that are often linked to prejudice and marginalization.

In Brazil, the Psychosocial Care Network (RAPS) guarantees access to comprehensive care and treatment for individuals experiencing mental distress. During the pandemic, services were reorganized to address the crisis; however, the demand for care exceeded the availability of qualified professionals. In response, psychotherapy treatment centers and private psychotherapists began offering focused treatments to help individuals cope with the impacts of the pandemic.

The severity of the pandemic coincided with a RAPS that was still in the process of organization and restructuring of community-based support services. Official documents published in Brazilian state capitals issued similar guidelines: (1) maintain services at Psychosocial Care Centers; (2) suspend collective activities, such as support groups; (3) implement hygiene measures; and (4) adopt social distancing. However, these documents largely failed to provide technical strategies to guide mental health professionals in managing cases during the crisis, focusing primarily on administrative measures (Weintraub, 2025). This study investigated the five largest psychotherapy centers in southern Brazil and highlights that, in the event of future pandemic scenarios, mental health treatment centers should aim to encompass the largest possible number of individuals in need of care. This would promote timely mental health support, strengthen suicide risk prevention, and foster a holistic approach to care that goes beyond a narrow focus on pathology or administrative issues (Kemper et al., 2015).

Prior to 2020, online psychotherapy was not formally recognized by the Brazilian Unified Health System (SUS) or by some health councils; therefore, psychotherapists had to quickly adapt to telehealth technologies. The global COVID-19 crisis brought this modality into focus, particularly in contexts associated with large-scale calamitous events, such as wars and pandemics (Castro et al., 2022). Therefore, after the pandemic, it is essential that mental health teams and policymakers critically assess and consolidate strategies developed during the pandemic, including telehealth and the use of social media. These approaches facilitated access to care and increased bonds between professionals, service users, and family members (Weintraub, 2025).

This study results indicate that satisfaction with social relationships had a protective effect, reducing the risk of life-threatening behavior by 26%. Although the sample size was relatively small (*n* = 94), this finding is relevant to public health, as it underscores the importance of continuity of care and strengthening social relationships as key strategies for coping with crisis situations. Mental health professionals working in community-based and territorial health services, who routinely provide care for patients with severe mental illness, are particularly concerned about the risk of life-threatening behavior in these populations (Rodrigues et al., 2024).

In Brazil, suicide deaths increased during the second year of the pandemic. In the Southeast, suicides among women increased by 28%, while increases of 32% were observed in the North region and 61% in the Northeast. In the Southeast, the most affected age group was 30–59 years, whereas in the North and Northeast, suicides were more prevalent among women aged 60 years or older. In contrast, in the South, suicides among women remained within the expected rates, regardless of age group or pandemic year (Orellana et al., 2024). In this study, 47% (*n* = 35) of women exhibited life-threatening behavior. To address this risk preventively, health services should prioritize strategies aimed at strengthening social relationships, thus increasing satisfaction with interpersonal bonds. Mental health teams can encourage discussion groups, identify community-level factors that contribute to psychological distress, and identify practical resources that can provide targeted care to specific vulnerable populations. Interventions designed to support social support networks or to develop protective resources for women exposed to violence, for example, are fundamental to sustain mental health benefits and prevent life-threatening behavior.

This study has some limitations. First, both social relationships and life-threatening behavior were evaluated using only one item from each instrument, selected to meet the specific objectives of this study. Consequently, depressive disorders and broader social factors were not comprehensively evaluated. Another limitation concerns the use of item nine of the PHQ-9, which has been questioned by some researchers as an insufficient tool to assess suicide risk and suicide ideation (Na et al., 2018). Nevertheless, more recent studies employing this same item in combination with machine-learning algorithms in large samples (*n* = 8,760) have demonstrated its reliability and usefulness as a screening tool for suicidal ideation in primary health care settings (Kim et al., 2021). Additionally, the relatively small sample size limited the statistical power required to detect significant differences across some variables, thereby restricting the depth of analysis. These factors constrain the generalizability of the findings. Furthermore, the study relied on a convenience sample, meaning that the results are only generalizable to samples similar to those of the participants included. Although one of the items addressed social support, this study could not specifically address the pulling-together effect, as no questionnaires addressing this topic were found. Moreover, we could not find any study examining the association between suicide risk and social relationships in patients undergoing online psychotherapy, suggesting that the outcomes could be related to the pulling-together effect. Finally, longitudinal studies are needed to clarify whether the protective effect of social relationships persisted during the pandemic.

We also highlight how government actions can expand access to mental health care for vulnerable populations. The prevention of life-threatening behavior must be interpreted as an urgent and ongoing public health priority (Gunnell et al., 2020). Although the pandemic initially represented an unknown context for many therapists, online psychotherapy emerged as a viable and effective alternative, contributing to the mental health care of vulnerable populations during post-crisis periods marked by widespread exhaustion. The prevention and promotion of mental health have therefore become urgent and require adaptations in the use of technology to reach populations living far from urban centers. Such strategies are especially relevant in the event of future pandemics, in which social isolation may once again be necessary.

## Conclusions

Our results suggest that satisfaction with social relationships had a protective effect against suicidal behavior during the early stages of the COVID-19 pandemic. Future longitudinal studies are needed to identify factors related to the pulling-together effect and to advance discussions on mental health promotion and disease prevention during global crisis.

## Supplementary Information

Supplementary Material 1.

## Data Availability

The raw data supporting the conclusions of this article will be made available by the corresponding author (Cleonice Zatti; cleonice.zatti@outlook.com) upon reasonable request, subject to restrictions arising from a research agreement with the five participating psychotherapy services.

## Abbreviations

COVID-19: Coronavirus disease 2019
HCPA: Hospital de Clínicas de Porto Alegre
FUMM: Fundação Mário Martins
HNSC: Hospital Nossa Senhora Conceição
HSP: Hospital São Pedro
CELG: Centro de Estudos Luís Guedes
PHQ-9: Patient Health Questionnaire-9
EUROHIS-QOL 8-item: Europe Health Interview Surveys Quality of Life Abbreviated Instrument
PR: Prevalence Ratio
RAPS: Psychosocial Care Network
