# Supplementary material for: Social support and life-threatening behavior in online psychotherapy patients: a possible “pulling-together effect” during COVID-19
Source: Psicol Reflex Crit. 2026 Mar 20;39:15. doi: 10.1186/s41155-026-00384-3 (PMC13057070; doi:10.1186/s41155-026-00384-3)
Supplement: Supplementary file 1 — Supplementary Material 1. [file 41155_2026_384_MOESM1_ESM.pdf]

TIKINET

CNPJ: 15.267.097/0001-70
R. Santanésia, 528 - 1º andar, Vila Pirajussara
CEP: 05580-055 | São Paulo – SP
(11) 2361-1808
www.tikinet.com.br

São Paulo, February 02, 2026

CERTIFICATE

TIKINET EDIÇÃO LTDA. – EPP, located at Avenida Vital Brasil, 466, Butantã, São Paulo-SP, 05503-000, Brazil, has qualified professionals to perform the services of copyediting and translation of texts in foreign languages. We attest that the following hired services were performed in their totality.

Type of Service: (X) Copyediting / (   ) Translation

Language: English

Document: SOCIAL SUPPORT AND LIFE-THREATENING BEHAVIOR IN ONLINE PSYCHOTHERAPY PATIENTS: A POSSIBLE "PULLING-TOGETHER EFFECT" DURING COVID-19

....

Carlos Eduardo Chiba
Tikinet Edição Ltda. – EPP
CNPJ: 15.267.097/0001-70
